# Synergistic Effect of the Long-Term Overexpression of Bcl-2 and BDNF Lentiviral in Cell Protecting against Death and Generating TH Positive and CHAT Positive Cells from MSC

**DOI:** 10.3390/ijms22137086

**Published:** 2021-06-30

**Authors:** Paulina Borkowska, Aleksandra Zielinska, Monika Paul-Samojedny, Rafał Stojko, Jan Kowalski

**Affiliations:** 1 Department of Medical Genetics, Medical University of Silesia, 41-200 Sosnowiec, Poland; azielinska@sum.edu.pl (A.Z.); mpaul@sum.edu.pl (M.P.-S.); genomika@sum.edu.pl (J.K.); 2 Chair and Department of Gynecology, Obstetrics and Oncological Gynecology, Medical University of Silesia, 40-211 Katowice, Poland; rstojko@sum.edu.pl

**Keywords:** lentiviral transduction, BDNF, Bcl-2, mesenchymal stem cells, cholinergic neurons, dopaminergic neurons, differentiation

## Abstract

Mesenchymal stem cells (MSC) are potentially a good material for transplantation in many diseases, including neurodegenerative diseases. The main problem with using them is the low percentage of surviving cells after the transplant procedure and the naturally poor ability of MSC to spontaneously differentiate into certain types of cells, which results in their poor integration with the host cells. The aim and the novelty of this work consists in the synergistic overexpression of two genes, *BCL2* and *BDNF*, using lentiviral vectors. According to our hypothesis, the overexpression of the *BCL2* gene is aimed at increasing the resistance of cells to stressors and toxic factors. In turn, the overexpression of the *BDNF* gene is suspected to direct the MSC into the neural differentiation pathway. As a result, it was shown that the overexpression of both genes and the overproduction of proteins is permanent and persists for at least 60 days. The synergistically transduced MSC were significantly more resistant to the action of staurosporine; 12 days after transduction, the synergistically transduced MSC had a six-times greater survival rate. The overexpression of the Bcl-2 and BDNF proteins was sufficient to stimulate a significant overexpression of the *CHAT* gene, and under specific conditions, the *TH*, *TPH1,* and *SYP* genes were also overexpressed. Modified MSC are able to differentiate into cholinergic and dopaminergic neurons, and the release of acetylcholine and dopamine may indicate their functionality.

## 1. Introduction

Mesenchymal stem cells (MSC) have great potential when it comes to using them in regenerative medicine, specifically in cell therapy [1,2,3]. Particularly high hopes have been placed on their use in treating neurodegenerative diseases [4,5]. A big advantage of MSC is the fact that they are easily isolated from various tissues, from Wharton’s Jelly (WJ-MSC) among others; they also have a low tumorigenic potential and are characterized by a high degree of plasticity [6]. Their low expression of MHC molecules makes it possible to use them in allogeneic transplants [7,8]. MSC easily differentiate into the different cell types from all three germ layers [6,9,10]. Many research groups have differentiated MSC into dopaminergic cells [11,12,13], which can be used to treat Parkinson’s disease. The ability of MSC to differentiate into cholinergic neurons [14] has also been described, as well as the transplant effect of the MSC cholinergic neurons, which resulted in improved spatial learning and memory ability in rats with Alzheimer’s disease [15]. However, the main problem that is associated with using progenitor or differentiated dopaminergic or cholinergic cells is their high mortality rate after transplantation.

Both native-undifferentiated MSC and in vitro differentiated MSC, e.g., into neural progenitors, are sensitive to the “stress” that is associated with the transplant procedure. In addition, by their basic nature, they do not spontaneously transform into functional nerve cells. As a result, transplant cells that have been weakened by the transplant procedure die in the foreign brain environment [16]. In an attempt to resolve these issues, a hypothesis was proposed to genetically modify MSC, which would render them resistant to any toxic factors, as well as giving them an increased ability to differentiate into nerve cells.

In order to obtain the above-mentioned results, two genes, which due to lentiviral transduction are simultaneously overexpressed in WJ-MSC, were selected. The Bcl-2 protein is a widely known anti-apoptotic factor [17,18,19]. The functional overexpression of the *BCL2* gene is expected to increase the Bcl-2 protein production, and thereby increase cell survival and their resistance to toxic factors [20,21,22]. As the BDNF protein in the brain stimulates neurogenesis [23] and synaptogenesis [24], it plays an important role in the creation of functional neurons. A functional overexpression of the *BDNF* gene is expected to increase the BDNF protein expression and thereby increase the possibility of the MSC differentiating into nerve cell progenitors. The aim of the experiment was to obtain cells that are resistant to the adverse effects of the environment and that are differentiated into neurons.

## 2. Results

**Figure 1:**
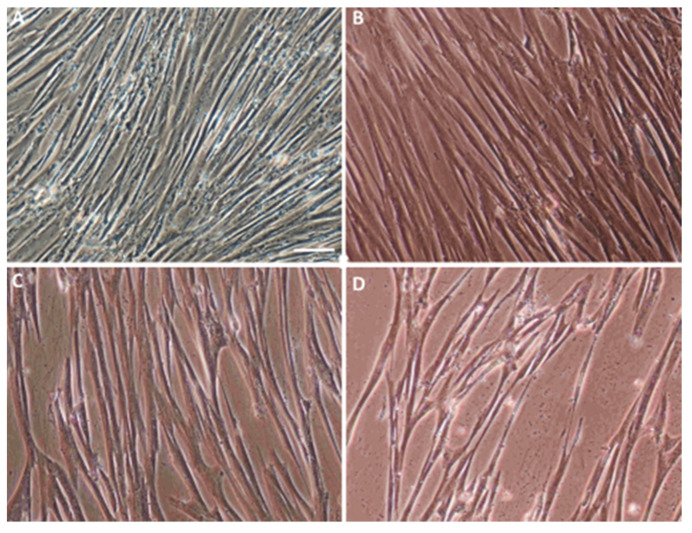
Characterization of an in vitro WJ-MSC culture. (**A**) Characteristic WJ-MSC fibroblast-like morphology. The WJ-MSC were cultured in a complete medium, as described in Materials and Methods. Changes in the cell morphology after a 12-day differentiation procedure in the control—C (**B**); empty vectors—EV (**C**); and full vectors—FV (**D**) group. Scale bar = 50 µm.

**Figure 2:**
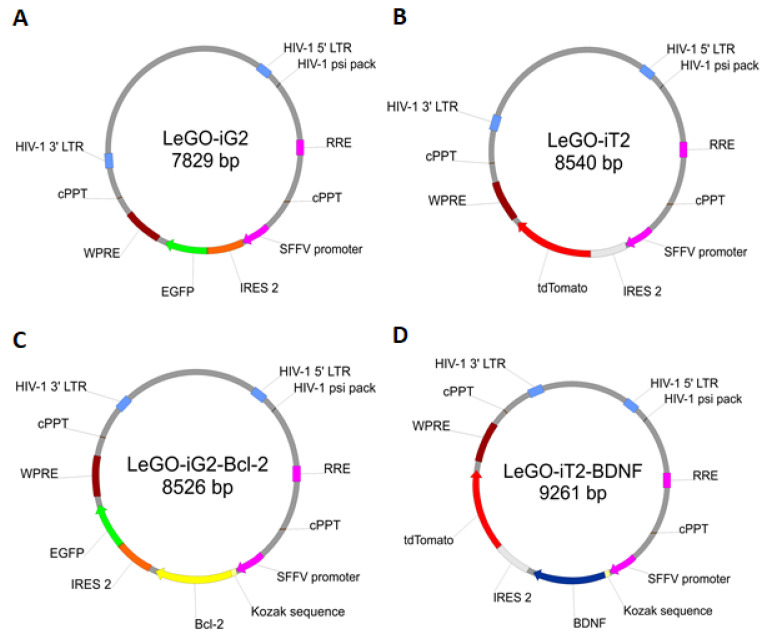
Lentiviral vector constructs. The lentiviral backbone plasmids contained the green fluorescence protein (EGFP) (**A**) or red fluorescence protein (tdTomato) (**B**). Into backbone A, coding sequence of *BCL2* gene (**C**), and into backbone B, coding sequence of *BDNF* gene (**D**), were cloned to overproduced Bcl-2 and BDNF proteins. In the further parts of this work, plasmids A and B were used synergistically for transduction under the abbreviation EV—empty vectors, and plasmids C and D under the abbreviation FV—full vectors.

All of the tests were conducted in three groups during the entire experiment:

(a) The control, marked with the working symbol (C), was the WJ-MSC culture. These cells were not subjected to lentiviral transduction or any additional substances. Their culture was carried out in parallel with the cultures of the cells from the other two groups in an analogous culture medium, but without any additional stimulators (Figure 1B).
(b) A control was transduced with the LeGO-iG2 and LeGO-iT2 “empty” vectors (EV) that contained the green or red fluorescent reporter proteins (Figure 2A,B) but no cloned genes that were to be overexpressed. That culture was carried out in parallel with the culture of the cells from the other two groups in an analogous culture medium, and the only additional stimulator was transduction with the empty skeletons of the lentiviruses (Figure 1C).
(c) The study group was transduced with a pair of LeGO-iG2-Bcl-2 and LeGO-iT2-BDNF vectors (full vectors—FV) in order to undergo a synergistic overexpression (Figure 2C,D). That culture was carried out in parallel with the culture of the cells from the other two groups in an analogous culture medium, and the only additional stimulator was *BCL2* and *BDNF* overexpression (Figure 1D). In some cases, the addition of bFGF or resveratrol was investigated, and then the group was indicated as FV + bFGF or FV + resveratrol.

### 2.1. Characterization of the Isolated WJ-MSC

The first step in implementing the research objectives described above was isolating the Wharton’s Jelly-derived mesenchymal stem cells (WJ-MSC) (Figure 1A). For the purpose of the preliminary tests, non-commercial, homogeneous lines WJ-MSC were isolated, evaluated, and banded. The WJ-MSC were labeled and it was confirmed that they were a homogeneous population of CD73 (+), CD90 (+), CD34 (-), CD11b (-), CD45 (+), and HLA-DR (-) cells. It was also confirmed that they could differentiate into adipocytes and osteoblasts. The results of these analyses are part of a published work on related topics [25].

### 2.2. Efficiency of WJ-MSC Transduction and Overexpressed Protein Levels

**Figure 3:**
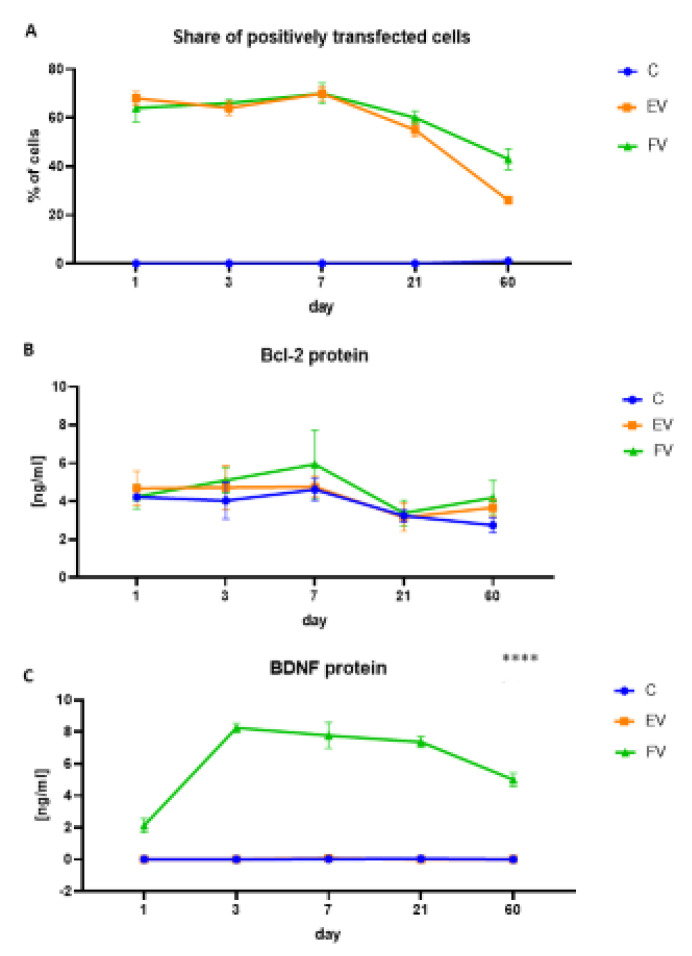
Transduction effects. Percentage of positive transduced cells over time (**A**). Quantitative analysis of the production of the Bcl-2 (**B**) and BDNF (**C**) proteins over time. Cells that were subjected to a synergistic vector transduction, which induced the overproduction of Bcl-2 and BDNF (full vectors—FV); empty vector transduced cells—(EV); control (**C**). In (**C**), the BDNF protein level of the EV group is the same as the C group (0 ng/mL all the time). The points represent the mean value (*n* = 6; three independent experiments). Statistically significant *p* < 0.05, **** *p* < 0.0001; two-way ANOVA test followed by a post hoc Tukey’s test.

Based on an assessment of the fluorescence level of the reporter proteins, the stability of the synergistic WJ-MSC transduction using a selected pair of the EV or FV vectors was determined. The concentration of the vectors in a given pair were selected to obtain an average level of transduction that was stable over time, and therefore the percentage of cells that was positively transduced in the FV and EV groups was similar. We achieved average infection rates, which means that about 60% of the cells were fluorescence positive (average for the entire experiment, measurements at five time points: t = 1; t = 3; t = 7; t = 21; t = 60: LeGO-iG2-Bcl-2 = 24% positive, LeGO-iT2-BDNF = 8% positive and both vectors = 30% positive). The highest infection rate was achieved on the 7th day of the experiment (LeGO-iG2-Bcl-2 = 20% positive, LeGO-iT2-BDNF = 9% positive and both vectors = 43% positive). The survival rate was determined based on the number of infected cells (Figure 3A). During the same period, i.e., more than 60 days, the expression level of the Bcl-2 (Figure 3B) and BDNF (Figure 3C) proteins was determined. There was an increased, but not statistically significant, overproduction of Bcl-2 protein in the FV group, compared to the EV or C groups. The highest overproduction was observed seven days after transduction. There was a clear and statistically significant overproduction of BDNF protein at the highest overproduction between days 3 and 21 after transduction (Figure 3C, t = 3, EV vs. FV: *p* < 0.0001; t = 7, EV vs. FV: *p* < 0.001; t = 21 EV vs. FV: *p* < 0.0001). The proteins were overproduced, and their level varied over time, but remained at a constantly elevated level relative to the control group (C), even 60 days after transduction (Figure 3C, t = 60, EV vs. FV: *p* < 0.001).

### 2.3. Cytoprotective Effect Depending on the Duration of BCL2 and BDNF Overexpression

**Figure 4:**
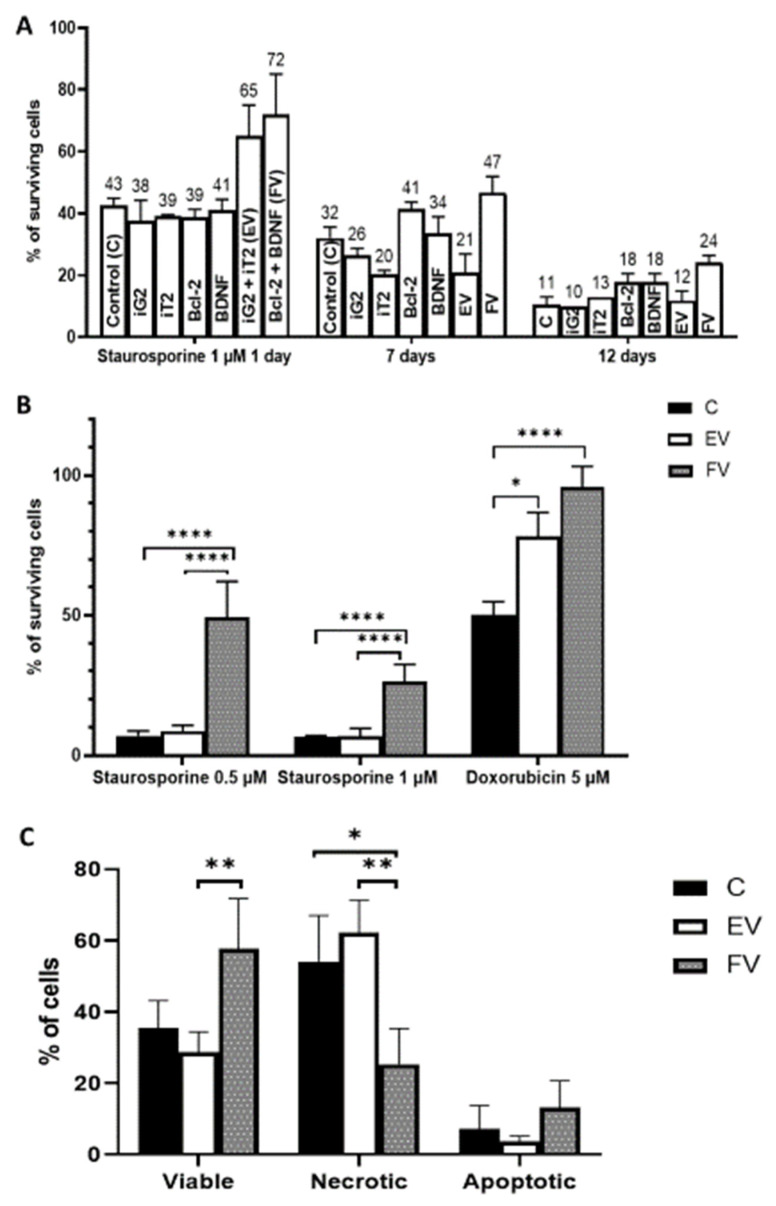
Cell survival analysis. Transduction using a single vector compared to the synergistic transduction of empty vectors (EV: iG2 + iT2) or full vectors (FV: *BCL2* + *BDNF*). Cell death was induced one day (A, Left) or seven days (A, Right) after the transduction. The WST-1 assay was carried out one day after the staurosporine was added; ordinary one-way ANOVA test (**A**). Cell survival analysis after various death inducers were used. Cell death was induced 12 days after transduction. The WST-1 assay was carried out 1 day after each agent was added; ordinary, one-way ANOVA followed by a post hoc Tukey’s test (**B**). An examination of cell resistance to a toxic agent and an analysis of its type of death. Cell death was induced 12 days after transduction. The measurement was conducted 12 h after 1 µM of staurosporine was added; Kruskal–Wallis test followed by post hoc Dunn’s multiple comparison test (**C**). The points represent the mean value ± SD (*n* = 12; three independent experiments). Statistically significant * *p* < 0.05, ** *p* < 0.01 or **** *p* < 0.0001.

The aim of this part of the experiment was to show that during the period of the overproduction of Bcl-2 and BDNF, the cytoprotective effect of their synergistic overexpression improved (Figure 4A, Left, one day after transduction, EV vs. FV: ns; Figure 4A, Middle, seven days after transduction, EV vs. FV: *p* < 0.0001; Figure 4A Right and Figure 4B, 12 days after transduction, EV vs. FV: *p* < 0.0001). From the study of cell survival that was performed, we additionally concluded that none of the vectors that were used individually had cytoprotective properties, in contrast to the synergistic application of the two vectors that caused the synergistic overexpression of the *BCL2* and *BDNF* genes (Figure 4A, Middle, C vs. FV: *p* < 0.01; FV vs. EV: *p* < 0.0001). In the next step (Figure 4A Right and Figure 4B), which was aimed at demonstrating the cytoprotective properties of the long-term synergistic overexpression of the *BCL2* and *BDNF* genes, the culture was continued for 12 days after the end of the transduction. As a result, a clear, statistically significant cytoprotective effect of the overexpression was demonstrated after using staurosporine to induce cell death by apoptosis via the intrinsic mitochondrial pathways (Figure 4B, staurosporine 0.5 µM and staurosporine 1 µM, EV vs. FV: *p* < 0.0001) [26]. In the case of doxorubicin (5 µM), we did not observe any differences between the EV and FV groups, so changes may have only been the effect of infection (Figure 4B, C vs. EV: *p* < 0.05), rather than a combination of Bcl-2 and BDNF overproduction (Figure 4B, EV vs. FV: ns). The results showed that the duration of the culture was lengthened in order to strengthen the positive cytoprotective effect of the Bcl-2 and BDNF overproduced proteins. After the short-term overexpression (Figure 4A Left) was compared with the long-term overproduction (Figure 4A Right and Figure 4B) of the investigated proteins: Bcl-2 and BDNF (FV), we could state that the long-term overexpression had a cytoprotective effect on the MSC under staurosporine conditions. A full post hoc analysis is presented in Table 1.

### 2.4. Analysis of Cell Death

An analysis was performed to determine which type of cell death is preferable after Bcl-2 and BDNF overexpression. The authors investigated if overexpression of Bcl-2 and BDNF proteins reduced cell death by necrosis (Figure 4C, EV vs. FV: *p* < 0.01). A smaller percentage of cells died by apoptosis (e.g., EV group: necrosis ~60% of cells vs. ~5% of cells), and significant effects of overexpression of Bcl-2 and BDNF proteins were not observed in the apoptotic cells (Figure 4C, EV vs. FV: *p* = 0.11).

### 2.5. Neuronal Gene Expression Level

**Figure 5:**
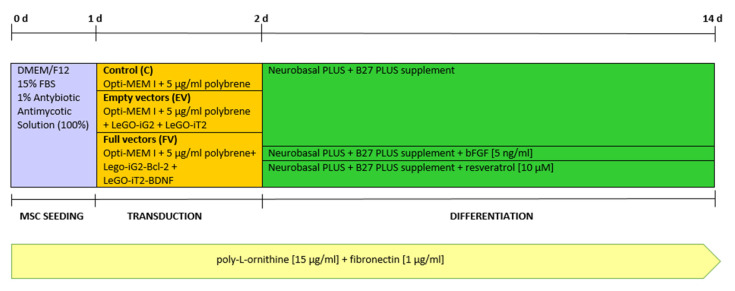
Overview of the methods for seeding, transducing, and differentiation.

**Figure 6:**
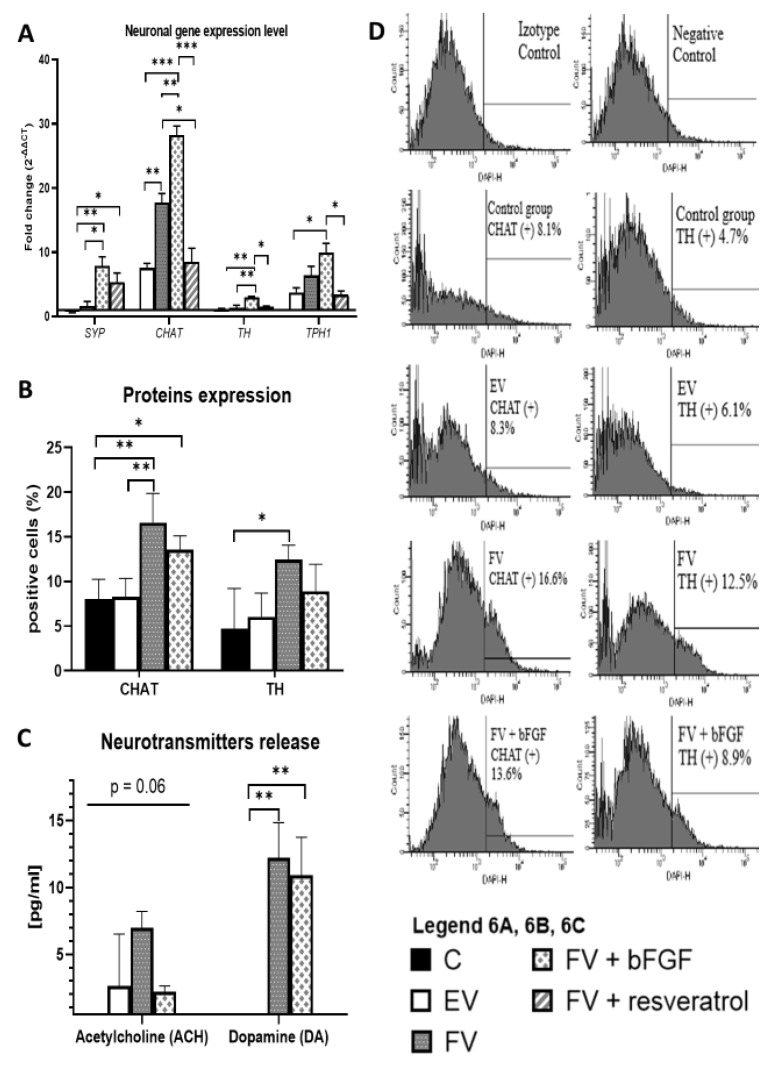
Differentiation effects. The RT-qPCR analysis for the neuronal markers is presented as the fold change (2^−ΔΔCT^) in the level of their expression, which was normalized to the *RPS17* reference gene. Statistically significant * *p* < 0.05; ** *p* < 0.01 or *** *p* < 0.001, ordinary one-way ANOVA followed by a post hoc Tukey’s test (**A**). The number of CHAT and TH positive cells. Calculation based on a flow cytometry analysis, presented as a % of positive stained cells from the total cell numbers. Points represent the mean value ± SD (*n* = 9; three independent experiments). Statistically significant * *p* < 0.05; ** *p* < 0.01, ordinary one-way ANOVA followed by a post hoc Tukey’s test (**B**). Acetylcholine and dopamine release after depolarization using the neuronal differentiated WJ-MSC. The points represent the mean value (*n* = 6; three independent experiments). Statistically significant * *p* < 0.05 or ** *p* < 0.01, ordinary one-way ANOVA test (**C**). Flow cytometric analysis for the expression of the neural specific proteins: CHAT (choline acetyltransferase) and TH (tyrosine hydroxylase) after 12 days of differentiation. Figure 6D shows results from one exemplary sample from each of the study groups. The authors intention was to illustrate how the exemplary histograms appeared and where the cut-off site was. The percentage values represent the mean values (*n* = 9; three independent experiments) and are presented as a bar chart in part B of this figure (**D**). Control group (C), the group that had been transduced with the empty vectors (EV), the group of cells that had overproduced the Bcl-2 and BDNF proteins (FV), and the FV that had additionally been supplemented with bFGF or resveratrol.

The WJ-MSC were differentiated toward cells with a neuronal phenotype (Figure 5). RNA from the cultured cells was isolated and the level of gene expression was analyzed in the control group (C), in the transduced empty vector (EV) group, in the group expressing Bcl-2 and BDNF (FV) proteins, and in the FV groups in combination with bFGF or resveratrol. The overexpression of the Bcl-2 and BDNF proteins was sufficient to stimulate a significant overexpression of the *CHAT* gene, which is typical of cholinergic (CHAT) neurons (Figure 6A, *CHAT*, EV vs. FV: *p* < 0.01). Adding bFGF during the differentiation procedure additionally increased the expression level of the *CHAT* (Figure 6A, *CHAT*, FV vs. FV + bFGF: *p* < 0.01), resulting in an increase in the expression level of the *TH* gene that is typical for dopaminergic neurons (Figure 6A, *TH*, FV vs. FV + bFGF: *p* < 0.01), and additionally, the *SYP* gene, which is involved in synaptogenesis process (Figure 6A, *SYP*, FV vs. FV + bFGF: *p* < 0.05). Synergistic overexpression and bFGF used together during the differentiation procedure increased the level of the *SYP* gene expression (Figure 6A, *SYP*, EV vs. FV + bFGF: *p* < 0.01) and at the same time increased the expression of the other genes in each investigated differentiation pathway: cholinergic (Figure 6A, *CHAT*, EV vs. FV + bFGF: *p* < 0.001), dopaminergic (Figure 6A, *TH*, EV vs. FV + bFGF: *p* < 0.01), and serotoninergic (Figure 6A, *TPH1*, EV vs. FV + bFGF: *p* < 0.05).

### 2.6. CHAT and TH Protein Expression

After the differentiation procedure, the cell numbers that expressed the proteins that are characteristic for the cholinergic (CHAT—choline acetyltransferase) and dopaminergic (TH—tyrosine hydroxylase) neurons were analyzed (Figure 6B,D). The synergistic overexpression of Bcl-2 and BDNF proteins caused a significant increase in the level of the expression of the neuronal proteins: CHAT (Figure 6B, CHAT, EV vs. FV: *p* < 0.01) and TH (Figure 6B, TH, C vs. FV: *p* < 0.05). Using bFGF in the differentiation procedure did not improve CHAT and TH expression (Figure 6B, CHAT and TH, FV vs. FV + bFGF: ns).

### 2.7. Release of the Neurotransmitters

The final stage of the preliminary research was depolarizing the differentiated cells and measuring the level of neurotransmitters that had been secreted as a result of the process. The level of acetylcholine and dopamine was also measured. The WJ-MSC produced an increased amount of the Bcl-2 and BDNF proteins (FV), then differentiated and secreted a greater level of acetylcholine compared to the cells from the other groups. The overexpression of the *BCL2* and *BDNF* genes (Figure 6C, DA, EV vs. FV: *p* < 0.01), as well as the use of bFGF, during the differentiation procedure (Figure 6C, DA, EV vs. FV + bFGF: *p* < 0.01) significantly decreased the ability of the obtained cells to release dopamine after the depolarization procedure was used.

## 3. Discussion

MSC derived from Wharton Jelly have been proposed as a promising cell source for the regeneration and restoration of neurodegenerative diseases. However, the poor survival rate of implanted cells hampers therapeutic efficacy. The goal of the authors was to synergistically overexpress two types of genes in WJ-MSC, and then to differentiate these WJ-MSC into neuron progenitors. One overexpressed gene, *BCL2,* is intended to increase the resistance of cells to toxic agents and to increase their survival rate in an adverse environment and, therefore, to be anti-apoptotic. In turn, the second gene, *BDNF,* is intended to intensify neurogenesis and synaptogenesis. A transplant has a better chance of survival if the cells that comprise it integrate with the host tissue and perform their appropriate functions [27]. Authors hypothesized that the overexpression of *BDNF* will change the modified MSC in such a way that, under the influence of the brain microenvironment, they will ultimately differentiate into specific neurons [28], and because of the overexpression of the genes that increasing synaptogenesis, it will be possible to create a network of neurons that will have conductivity.

Although other authors have investigated the effects of the overexpression of individual *BCL2* [29,30,31] or *BDNF* [12,32,33] genes on increased survival rate or ability of MSC to differentiate into neuron-like cells, no group of researchers has yet studied the effects of the synergistic overexpression of two genes, *BCL2* and *BDNF*, on developing a resistance to toxic agents and the ability to differentiate MSC into neuron progenitors.

In this experiment, the stability of their synergistic overexpression was studied for the first time. It was shown that transduction was stable over time, even 60 days after the transduction was performed, and the number of transduced cells was significantly higher compared to the control group. The stability of this overexpression also translates into increasing the stability of the overproduction of BDNF protein. In the case of Bcl-2 protein, overexpression results in a slight increase in the amount of this protein, but the difference is not statistically significant. The obtained results are extremely important in terms of testing the genetically modified cells obtained in animal models for treating neurodegenerative diseases. After a transplant, transduction must be stable long enough to enable the cells to adapt to the new environment and to ultimately differentiate into a specific cell type, integrate with the host tissue, and perform their appropriate functions [34,35].

In the next stage of the experiment, the cytoprotective properties of transduction were investigated. The vectors were shown to have an additive effect after the single-vector transduction and under high toxicity conditions using 1 µM staurosporine. The synergistic cytoprotective effect of the two empty vectors (EV) was approximately the sum of the empty vectors applied individually. The cell was not indifferent to transduction of lentiviruses with empty backbones. The cell membrane was exposed to polybrene and the empty vector was not in fact empty, because it contained the genes encoding green or red fluorescence, which were expressed. Thus, transduction with empty lentiviruses affects the cell, in this case in a cytoprotective way. This is a notable effect generated by the procedure, and this phenomenon was also observed by other authors [36]. Similar relationships were observed in the case of full vectors (FV). The extension of the cultivation time after the transduction increased the amount of Bcl-2 and BDNF proteins, which made it possible to confirm that WJ-MSC, with a synergistic overexpression of both genes, had a significantly increased resistance to toxic factors. Previous results showed that the overexpression of the *BCL2* gene, and as a result, the over-production of the highly anti-apoptotic Bcl-2 protein increases the cell survival rate after toxic agents are used [30,31]. For this experiment, the ELSA test did not confirm a statistically significant overproduction of Bcl-2 protein. On the one hand, one problem for measuring the relative amount of overproduced protein could be the imperfection of the method. A similar problem, but with confirming BDNF overproduction using ELISA, was previously described [33]. On the other hand, there could have been a low overproduction of the Bcl-2 protein, which was statistically insignificant, but was sufficient to induce the biological effect of the cytoprotection of the MSC cells under toxic conditions (Figure 4A, Right, iG2 vs. Bcl-2: *p* < 0.05). Although the anti-apoptotic role of BDNF has also been demonstrated in the work of other researchers [37,38,39], no one has yet studied the synergistic effect of both proteins on cell survival rate.

When studying the properties of genetically modified MSC, an important aspect is to investigate the preferred pathway of cell death. The obtained results proved a significantly lower mortality rate for the cells with Bcl-2 and BDNF proteins overproduction. The decrease in the number of cells that were directed to the death pathway under the influence of the stressor staurosporine confirms the hypothesis that cells producing increased amount of Bcl-2 and BDNF have a greater chance of surviving the stress-inducing transplant procedure [40]. On the other hand, reducing the tendency of cells to die via necrosis, for the benefit of death by apoptosis, also reduces the tendency for the inflammatory process to occur at the transplant site, which increases the transplant’s chance of integrating with the host tissue [41].

The RT-PCR analysis showed that the overexpression of the studied genes (*BCL2* and *BDNF*) significantly increased the expression level of the genes that are typical for the cholinergic (*CHAT*) neurons. Earlier experiments confirmed the possibility of differentiating MSC into cholinergic neurons [42,43] or for increased synaptogenesis [34]. In line with the results of other researchers, the present experiment confirmed that the use of bFGF during the differentiation procedure causes an increase in the expression of the *TH* genes that are typical for the dopaminergic neurons [12,13], while there was an increase in the expression of *CHAT* and *TPH1* genes with a simultaneous increase in the expression of the synaptophysin (*SYP*) gene. The addition of resveratrol did not result in better differentiation effects than using empty vector (EV) transduction. There was a positive effect of resveratrol only in the case of the increased expression of the synaptophysin gene; however, the positive effect of resveratrol on the synaptogenesis process has already been demonstrated by other researchers [44]. Increasing the expression of the genes that are characteristic of various neurons may suggest that the cells obtained in the process of differentiation are a heterogeneous population of nerve cells. The in vivo studies based on this experiment will be carried out according to the following scheme: the MSCs will be transduced in vitro, then the modified MSC will be transplanted; followed by the differentiation process, which will be driven by overexpressing proteins and the brain microenvironment, and will occur in the brain [45,46]. Obtaining various kinds of neural cells in an in vitro experiment seems to be a beneficial solution, because it increases the plasticity of the obtained model and provides the opportunity for its further testing in various animal models of neurodegenerative diseases [47].

In addition to the increase in the level of gene expression, the overexpression of the Bcl-2 and BDNF proteins caused an increase in the expression of the CHAT (cholinergic neurons) and TH (dopaminergic neurons) proteins. After they are depolarized, the resulting cells are capable of secreting acetylcholine and dopamine, which may suggest their functionality. The mere overexpression of the *BCL2* and *BDNF* genes results in the production of a mixture of functional cholinergic and dopaminergic neurons in a culture, and possibly other types of neurons as well. As in the case of other researchers, as a result of the proposed protocol, a mixture of nerve cells was obtained [48,49], among which the population of a specific type of cells, be it cholinergic or dopaminergic, fluctuate at around 15% of the positive stained cells from the total cell numbers (Figure 6B, CHAT and TH, FV).

The novelty of the proposed differentiation model consists in the fact that, due to the overexpression of the *BCL2* and *BDNF* genes, the cells become much more resistant to toxic factors. In addition, gene overexpression is sufficient to direct cells to the neural differentiation pathway. Based on this preliminary research, it is worth performing a graft experiment. As different types of neurons were obtained in the experiment, the proposed protocol can be further investigated in in vivo experiments in various models of neurodegenerative diseases, because apart from gene overexpression the final role in determining the direction of differentiation is played by the brain’s microenvironment.

## 4. Materials and Methods

### 4.1. Isolating, Culturing, and Characterizing the WJ-MSC

The WJ-MSC were isolated from human umbilical cord and afterwards characterized according to a previously published protocol [25]. The study was approved by the Bioethical Committee of the Medical University of Silesia in Katowice (Resolution No. KNW/0022/KB/195/14), and all methods were performed in accordance with the relevant guidelines and regulations. The participants (mothers) were informed of the procedure in writing and gave their written consent to use the umbilical cords. The homogeneity of the WJ-MSC was quantified using flow cytometry, and the presence of CD73, CD90, CD34, CD11b, CD19, CD45, and HLA-DR was determined. The WJ-MSC were characterized based on their differentiation capacity toward adipocyte and osteocyte cells [25].

### 4.2. Lentiviral Vectors

In order to achieve the overexpression of the two genes equally, the lentiviral constructs were prepared according to a previously published protocol [25]. Thanks to the cloning to the empty backbones, LeGO-iG2 (Figure 2A) and LeGO-iT2 (Figure 2B) coding sequence of *BCL2* and *BDNF* genes, LeGO-iG2-Bcl-2 (Figure 2C) and LeGO-iT2-BDNF (Figure 2D), vectors were obtained that would induce overproduction of the Bcl-2 and BDNF proteins by the WJ-MSC. Additionally, in order to increase the functionality of that overexpression and increase the number of proteins that were produced in the vicinity of the START codon of each gene, the Kozak sequence was added.

### 4.3. Flow Cytometry Analysis

Based on the fluorescence level of the reporter proteins, EGFP in LeGO-iG2 backbone or tdTomato in LeGO-iT2 backbone, the effect and the stability of the transduction was determined. LeGO-iG2 was a gift from Boris Fehse (Addgene plasmid #27341; http://n2t.net/addgene:27341; RRID:Addgene_27341) and LeGO-iT2 was a gift from Boris Fehse (Addgene plasmid #27343; http://n2t.net/addgene:27343; RRID:Addgene_27343) [50]. The cells after transduction and culture (culture time was varied from 1 day to 60 days) were treated with 0.25% trypsin/EDTA (PAN-Biotech GmbH, Aidenbach, Germany; P10-029500) for 4 min to form single cells and then suspended in phosphate buffered saline (DPBS) (PAN-Biotech GmbH, Aidenbach, Germany; P04-36500). After, flow cytometry (BD FACSAria II; BD FACSDiva Software V6.1.2) was used to count the positively transduced cells in each groups. Positive cells were counted as the number of fluorescence-positive cells out of the total cells.

### 4.4. WJ-MSC Transduction, Protein Extractions

The WJ-MSC were plated one day prior to the transduction (200,000 cells per culture dish Ø 35 mm) in order to achieve a 70–80% confluence by the time of the transduction. The transduction was performed via a 24-h incubation of the cells with an optimal dilution of a virus in an Opti-MEM I Reduced Serum Medium (Life Technologies, Waltham, MA USA; 31985070) in the presence of 5 μg/mL polybrene. After two days of culture in a normal medium DMEM/F12 (PAN Biotech GmbH, Aidenbach, Germany; P04-41250) + 15% FBS (PAN Biotech GmbH, Aidenbach, Germany; P30-8500) and a 1% Antibiotic Antimycotic Solution (100×) (PAN Biotech GmbH, Aidenbach, Germany; P06-07300), the medium was replaced by a medium with a reduced FBS (2%). The medium was changed twice a week for 60 days. Cell lysis was performed on culture days 1, 3, 7, 21, and 60 to determine the amount of Bcl-2 and BDNF proteins that had been produced. The cells were trypsynized, pelleted, and lysed in 200 µL of RIPA buffer with 10% protease inhibitor cocktail (Sigma-Aldrich, Saint Louis, MO, USA; P8849-1ML).

### 4.5. ELISA Analysis of the Bcl-2 and BDNF Proteins

The concentration of protein in the cultured cells was measured using an ELISA human immunoassay kit: Human Bcl-2 ELISA Kit (Elabscience, Wuhan, China, catalog no. E-EL-H0114) and Quantikine ELISA Human BDNF Immunoassay (R&D Systems, McKinley Place, MN, USA catalog no. DBD00). The absorbance was read at 450 nm using an ELX 800 IU automated Microplate Reader (Bio-Tek Instruments, Inc., Winooski, VT, USA; Gene 5 Software V 3.02). The results were analyzed using a quadratic log–log curve fit.

### 4.6. Cell Viability WST-1 Colorimetric Assay

A WST-1 (4-(3-(4-iodophenyl)-2-(4-nitrophenyl)-2H-5-tetrazolio)-1,3-benzene disulfonate; (Roche Applied Science, Penzberg, Germany; 11644807001) colorimetric assay was performed to determine the effects of the Bcl-2 and BDNF proteins on cell viability. The assay was performed using 96-well plates with 5000 cells seeded in each well. The transduction was performed as described above, followed by a further cultivation in medium with reduced FBS for one day, seven days, or 12 days. Furthermore, the concentration-dependent (0.5 and 1 μM) effect of staurosporine (Sigma-Aldrich, Saint Louis, MO, USA; S4400) and (5 μM) doxorubicin (Sigma-Aldrich, Saint Louis, MO, USA; D1515) over a 24-h treatment was examined. To sum up and clarify, as in Figure 4A, cells were transduced, cultured for one day or seven days, and after apoptosis was induced. As shown in Figure 4B, cells were transduced, cultured for 12 days, and after apoptosis was induced. Time of culture was lengthened to strengthen the positive cytoprotective effect of the transduced cells.

After one day of treatment with an agent, a 100 µL 10% solution of WST-1 in DMEM/F12 w/o phenol red (PAN Biotech GmbH, Aidenbach, Germany; P04-41650) was added to the culture medium and incubated for 45 min at 37 °C. The absorbance was read at 450 nm using an ELX 800 IU automated Microplate Reader (Bio-Tek Instruments, Inc., Winooski, VT, USA). The results were analyzed using Bio-Tek Gene 5 Software V 3.02.

### 4.7. Cell Death Analysis

The assay was performed in 24-well plates with 50,000 cells seeded in each well. The transduction was carried out as described above, followed by a further cultivation in a medium with reduced FBS for 12 days. Then, the resistance to 1 μM staurosporine for 12 h and the manner in which the cells died after its application were examined. The cells were trypsynized, centrifuged (1200 rpm, 5 min, 4 °C), and washed once in PBS. Subsequently, 1 × 10^6^ cells per 1 mL were stained using a Vybrant DyeCycle Violet/SYTOX AADvanced Apoptosis Kit (Violet Chromatin Condensation/Dead Cell Apoptosis Kit; Life Technologies, Waltham, MA USA; A35135). Flow cytometry (BD FACSAria II; BD FACSDiva Software V6.1.2) was then used to analyze the results.

### 4.8. Neuronal Differentiation

The assay was performed in 12-well plates with 50,000 cells seeded in each well. All of the differentiation procedures were carried out in poly-L-ornithine and fibronectin-coated vessels. Immediately after transduction, the transduction medium was changed to a differentiating medium. In order to induce neuronal differentiation, a medium containing Neurobasal PLUS (Gibco/Thermo Fisher Scientific, Waltham, MA, USA; A3582901) and B27 PLUS supplement (Gibco/Thermo Fisher Scientific, Waltham, MA, USA: A3582801) was used. For the WJ-MSC cells that had been transduced by the “full vectors”, the neuronal differentiation medium was additionally supplemented with bFGF (5 ng/mL) (Gibco/Thermo Fisher Scientific Waltham, MA, USA; PHG0264) or resveratrol (10 µM) (Sigma-Aldrich, Saint Louis, MO, USA; 554325). The differentiation protocol was carried out for 12 days (Figure 2).

### 4.9. RT-qPCR

Fresh cells after differentiation were used to extract RNA [25]. The total cellular RNA was extracted using NucleoZOL (Macherey-Nagel GmbH, Dueren, Germany; 740404.200), according to the manufacturer’s instructions [25]. The primers for the selected genes, including *RPS17* (reference gene), *SYP*, *CHAT*, *TH*, and *TPH1*, were purchased from Sigma Aldrich (KiCqStart SYBR Green Primers) (Table 2). The one-step RT-qPCR was performed using a GoTaq 1-Step RT-qPCR System (Promega GmbH, Walldorf, Germany; A6020) [25]. The RT-qPCR protocol was a 15-min RT reaction at 37 °C, a 10-min PCR activation at 95 °C and then 40 cycles of a 10 s denaturation at 95 °C, a 30 s annealing at 60 °C at the lowest primer pair’s melting temperature, and a 30 s PCR extension at 72 °C [25]. Finally, a melting-curve analysis was performed to confirm the RT-qPCR specificity [25]. Negative controls with no total RNA were included in each run of the RT-qPCR [25]. Ct was automatically determined using Bio-Rad CFX Manager Software (Version 3.1) on a Bio-Rad CFX96 Real-Time System [25]. For the fold change (2^−ΔΔCT^) calculation, a control group was used cells which were not subjected to lentiviral transduction or any additional substances. Control culture was carried out in parallel with the cultures of the cells from the other groups in an analogous culture medium but without any additional stimulators. *RPS17* was used as a reference gene.

### 4.10. Evaluating the TH and CHAT Expression of the Proteins

After differentiation, the cells were washed twice in PBS and fixed in PBS with 4% paraformaldehyde (Sigma-Aldrich, Saint Louis, MO, USA; 158127). Then, the cells were washed three times in PBS with 1% BSA (Sigma-Aldrich; A9418), permeabilized with 0.3% Triton X-100 (Sigma-Aldrich, Saint Louis, MO, USA; T8787)/1% BSA/PBS with 5% normal goat serum (Jackson Immuno Research, Cambridge, UK; 005-000-121) for 45 min, and then incubated with rabbit anti-tyrosine hydroxylase antibody (dilution 1:600; AssayBioTech, Fremont, CA, USA; B0037) or with rabbit anti-choline acetyltransferase antibody (dilution 1:300; Proteintech, Manchester, UK; 20747-1-AP) in 1% BSA/PBS with 5% goat serum overnight at 4 °C. Subsequently, the cells were washed three times in PBS with 1% BSA and incubated with goat anti-rabbit IgG secondary antibody (DY405) (dilution 1:600; LSBio, Seattle, WA, USA; LS-C355899) in 1% BSA/PBS for 1 h in the dark at room temperature. Similar staining was done using a rabbit IgG isotype control (dilution 1:1500; Bioss, Woburn, MA, USA; bs-0295P) to determine any nonspecific binding. The cells were analyzed using flow cytometry (BD FACSAria II; BD FACSDiva Software V6.1.2).

### 4.11. Neurotransmitters Release Analysis

The assay was performed in 12-well plates with 50,000 cells seeded in each well. After transduction, differentiation was carried out for 12 days. Then, the cells were washed twice in PBS, after which the medium was replaced with a low K^+^ solution, Neurobasal-A (5.33 mM KCl) (Life Technologies, Waltham, MA USA; 10888022) for 30 min in a 250 µL/well. Next, the low K^+^ medium was collected and 4 mM of Sodium Metabisulphite (Sigma-Aldrich, Saint Louis, MO, USA; PHR1434) and 1 mM of EDTA (Sigma-Aldrich, Saint Louis, MO, USA; E7889) was added. When the medium was collected, the wells were replaced with a high K^+^ medium, Neurobasal-A medium, to which KCl had been added (Sigma-Aldrich, Saint Louis, MO, USA; P9541), which was required to obtain a total of 53–56 mM KCl. Afterward, the cells were incubated for 30 min in a 250 µL high K^+^ solution/well. At the end of the termination period, the high K^+^ medium was collected and 4 mM of Sodium Metabisulphite and 1 mM of EDTA were added. Both media (low and high K^+^) were centrifuged at 13,000 rpm for 15 min at 4 °C. The levels of the neurotransmitters (dopamine and acetylcholine) in the supernatant were immediately determined. The neurotransmitter levels were acquired and quantitated using an ELISA kit (Elabscience, Wuhan, China, catalog no. dopamine E-EL-0046; acetylcholine E-EL-0081), according to the manufacturer’s instructions. The absorbance was read at 450 nm using an ELX 800 IU automated Microplate Reader (Bio-Tek Instruments, Inc., Winooski, VT, USA; Gene 5 Software V 3.02). The supernatants (low and high K^+^) for both wells were analyzed. The amount of neurotransmitter produced per well was calculated based on the difference between the amount in a high K^+^ supernatant and the amount in a low K^+^ supernatant, in order to minimize the effect of the difference in the number of cells in the wells.

### 4.12. Statistical Analysis

The data are presented as the mean ± SD. The Shapiro–Wilk test for normal distribution was used. A two-way ANOVA, ordinary one-way ANOVA followed by a post hoc Tukey’s test, or Kruskal–Wallis test followed by a post hoc Dunn’s multiple comparison were used. The data were analyzed using GraphPad Prism software version 8.0. *p* < 0.05 was considered to be statistically significant. The fold change (2^−ΔΔCT^) method was used to the present RT-qPCR results.

## 5. Conclusions

The described experiment showed that the synergistic overexpression of the *BCL2* and *BDNF* genes, which was stable and functional over time:

1. increased the survival rate of the transduced cells under toxic conditions
2. directed the transduced cells into the neuronal differentiation pathway
3. indicated that the cells that are obtained as a result of the process produce neurotransmitters, and thus one can infer their functionality.

## Figures and Tables

**Table 1:** Ordinary one-way ANOVA followed by a post hoc Tukey’s test to Figure 4A. Statistically significant * *p* < 0.05, ** *p* < 0.01, *** *p* < 0.001 or **** *p* < 0.0001.

	Figure 4A Left			Figure 4A Middle			Figure 4A Right		
Tukey’s Multiple Comparisons Test	Significant?	Summary	Adjusted *p* Value	Significant?	Summary	Adjusted *p* Value	Significant?	Summary	Adjusted *p* Value
Control (C) (1d) vs. iG2 (1d)	No	ns	0.9834	No	ns	0.9847	No	ns	>0.9999
Control (C) (1d) vs. iT2 (1d)	No	ns	0.9969	No	ns	0.3946	No	ns	0.7552
Control (C) (1d) vs. Bcl-2 (1d)	No	ns	0.995	No	ns	0.3541	No	ns	0.1392
Control (C) (1d) vs. BDNF (1d)	No	ns	>0.9999	No	ns	0.9973	No	ns	0.1553
Control (C) (1d) vs. iG2+iT2 (EV) (1d)	Yes	*	0.0309	No	ns	0.6398	No	ns	0.9997
Control (C) (1d) vs. Bcl-2+BDNF (FV) (1d)	Yes	**	0.0036	Yes	**	0.0049	Yes	****	<0.0001
iG2 (1d) vs. iT2 (1d)	No	ns	>0.9999	No	ns	0.8561	No	ns	0.9566
iG2 (1d) vs. Bcl-2 (1d)	No	ns	>0.9999	Yes	*	0.0488	No	ns	0.5076
iG2 (1d) vs. BDNF (1d)	No	ns	0.9984	No	ns	0.7783	No	ns	0.5348
iG2 (1d) vs. iG2+iT2 (EV) (1d)	Yes	*	0.0121	No	ns	0.976	No	ns	>0.9999
iG2 (1d) vs. Bcl-2+BDNF (FV) (1d)	Yes	**	0.0017	Yes	***	0.0002	Yes	****	<0.0001
iT2 (1d) vs. Bcl-2 (1d)	No	ns	>0.9999	Yes	***	0.0006	No	ns	0.9783
iT2 (1d) vs. BDNF (1d)	No	ns	>0.9999	No	ns	0.0926	No	ns	0.9832
iT2 (1d) vs. iG2+iT2 (EV) (1d)	Yes	*	0.0178	No	ns	0.9992	No	ns	0.8891
iT2 (1d) vs. Bcl-2+BDNF (FV) (1d)	Yes	**	0.0024	Yes	****	<0.0001	Yes	**	0.0054
Bcl-2 (1d) vs. BDNF (1d)	No	ns	0.9998	No	ns	0.6515	No	ns	>0.9999
Bcl-2 (1d) vs. iG2+iT2 (EV) (1d)	Yes	*	0.0161	Yes	**	0.002	No	ns	0.2446
Bcl-2 (1d) vs. Bcl-2+BDNF (FV) (1d)	Yes	**	0.0022	No	ns	0.406	No	ns	0.0602
BDNF (1d) vs. iG2+iT2 (EV) (1d)	Yes	*	0.0314	No	ns	0.2117	No	ns	0.2688
BDNF (1d) vs. Bcl-2+BDNF (FV) (1d)	Yes	**	0.0042	Yes	*	0.0135	No	ns	0.0528
iG2+iT2 (EV) (1d) vs. Bcl-2+BDNF (FV) (1d)	No	ns	0.9307	Yes	****	<0.0001	Yes	****	<0.0001

**Table 2:** Information about the evaluated reference control genes and neuronal marker genes. The primer pair ID was compatible with the KiCqStart Primers (Sigma-Aldrich). Nomenclature according to HUGO Gene Nomenclature Committee.

	Markers	Gene Name	Primer Pair ID	Forward Primer	Reverse Primer
*RPS17*	endogenous control	Ribosomal Protein S17	H_RPS17_1	CCATTATCCCCAGCAAAAAG	GAGACCTCAGGAACATAATTG
*TH*	dopaminergic neurons	Tyrosine Hydroxylase	H_TH_1	CAAAATCCACCATCTAGAGAC	CTGACACTTTTCTTGGGAAC
*CHAT*	cholinergic neurons	Choline O-Acetyltransferase	H_CHAT_1	TCATTTCTTTGTCTTGGATG	TGGAAGCCATTTTGACTATC
*TPH1*	serotoninergic neurons	Tryptophan Hydroxylase 1	H_TPH1_1	AAAGAGCGTACAGGTTTTTC	GTCTCACATATTGAGTGCAG
*SYP*	synaptogenesis marker	Synaptophysin	H_SYP_1	CCCTTCGGTATTGTTCAAAG	TTTGACTAGGTGGTTAAGGAG

## Data Availability

The data that support the findings of this study are available from the corresponding author upon reasonable request.
